# Oncoplastic surgery for Paget’s disease of the breast

**DOI:** 10.3389/fonc.2023.1151932

**Published:** 2023-05-17

**Authors:** Rafael José Fábio Pelorca, Idam de Oliveira-Junior, René Aloisio da Costa Vieira

**Affiliations:** ^1^ Programa de Pós-Graduação em Tocoginecologia, Faculdade de Medicina de Botucatu, Botucatu, SP, Brazil; ^2^ Programa de Pós-Graduação em Oncologia, Barretos Cancer Hospital, Barretos, SP, Brazil; ^3^ Departamento de Mastologia e Reconstrução Mamária, Barretos Cancer Hospital, Barretos, SP, Brazil; ^4^ Departamento de Cirurgia Oncológica, Divisão de Mastologia, Hospital de Câncer de Muriaé, Muriaé, MG, Brazil

**Keywords:** Paget’s disease, mammary, breast neoplasms, breast reconstruction, plastic surgery, oncoplastic surgery

## Abstract

**Introduction:**

Paget’s disease of the breast (PDB) is a rare nipple entity associated with multifocality. Due to its location, resection of the entire nipple-areolar complex is necessary. Historically central quadrantectomy and mastectomy have the surgical treatments of choice. The feasibility of oncoplastic breast surgery (OBS) for PDB is unknown.

**Methods:**

This was a retrospective study performed in a Brazilian oncological hospital. We evaluated the factors related to the performance of OBS in PDB. In addition, the impact of OBS on local recurrence and survival was analysed. Comparisons were made between groups using the chi-square test, Mann−Whitney U test, and Kaplan–Meier method. To assess the impact factor of the variables on the performance of OBS, logistic regression was performed.

**Results:**

Eighty-five patients were evaluated. OBS was performed in 69.4% (n=59), and of these, 16 (27.2%) were symmetrized with contralateral surgery. Mastectomy without reconstruction was performed in 28.3% of the patients. The primary procedure performed was mastectomy with reconstruction (n=38; 44.7%), and the preferential technique for immediate reconstruction was skin-sparing mastectomy with prosthesis; for late reconstruction, the preferred technique was using the latissimus dorsi. Breast conserving-surgery was performed in 27.0% (n=23), primarily using the plug-flap technique (OBS). Age was associated with the use of OBS; as patients aged 40-49 exhibited a higher rate of OBS (p = 0.002; odds ratio 3.22). OBS did not influence local recurrence (p=1.000), overall survival (p=0.185), or cancer-specific survival (p=0.418).

**Conclusion:**

OBS improves options related to surgical treatment in PDB without affecting local recurrence or survival rates.

## Introduction

The surgical treatment of breast cancer has changed radically in the last two decades, with improvements in mastectomy, breast-conserving surgery (BCS) and axillary preservation. The oncological safety of BCS has been extrapolated to larger tumours, provided a favourable breast/tumour ratio is maintained (1, 2). Likewise, indications for neoadjuvant chemotherapy have increased the rates of BCS (3), and when mastectomy is indicated, immediate reconstruction using implants or even myocutaneous flaps has become common practice (4).

In this context, oncoplastic breast surgery (OBS) has recently emerged (5), in which plastic surgery techniques are added to the therapeutic arsenal for the treatment of breast cancer. Thus, another dimension in the approach to the breast/tumour relationship has been created, expanding the indications for BCS (2), even for larger tumours, thus giving rise to the concept of extreme oncoplasty (1). In the case of mastectomies, immediate reconstruction with myocutaneous flaps was replaced by implants, which is associated with shorter surgical duration, lower complication rates and easier performance (6, 7). Thus, despite conceptual questioning, some authors have begun to consider OBS techniques both for BCS (2, 8) and breast reconstruction (6, 9, 10). As breast surgeons become qualified, the range of surgical options will expand, improving patient quality of life (11) with no increase in the risk of recurrence (12, 13).

Due to its central location, the surgical treatments for Paget’s disease of the breast (PDB) have also been modified with OBS, which allows the use of different technical options (14). Patients initially were submitted to BCS with purse-string suturing or spindle incision, but now with OBS (14–16), patients are treated with local skin flaps using the plug-flap technique or with pedicle surgery or other techniques, which prepare the areolar region for future tattooing (16, 17). If the patient demonstrates indications for mastectomy, skin-sparing mastectomy with immediate reconstruction with a prosthesis (16) is one possibility and presents with good aesthetic results (4).

However, the spectrum of OBS techniques performed for PDB is unknown (18) given the rarity of this pathology and the need for a team trained in OBS. Few published studies have described OBS for PD (19, 20). Accordingly, we sought to evaluate this relationship in an oncology referral service where OBS is systematically performed.

## Materials and methods

This retrospective study was approved by the institutional Research Ethics Committee under numbers 657293 and CAAE 31046314.5.0000.5437. Patients with PDB treated at a tertiary cancer hospital between 2000 and 2021 were evaluated.

The patients were selected based on the presence of Paget’s disease in the surgical specimen of the breast according to the pathological database of the institution. The clinicopathological and surgical data of the patients, as well as data on local recurrence and survival, were obtained from the medical records and evaluated.

To evaluate tumour size, the total size of the tumour was considered, regardless of the associated *in situ* or invasive component. Likewise, in the molecular subtype analysis, the invasive component was evaluated, and in its absence, the ductal carcinoma *in situ* component was evaluated.

We sought to evaluate aspects related to surgery in PDB, particularly the use of oncoplasty techniques. We refer to oncoplastic breast surgery (OBS) for techniques used for breast-conserving surgeries (8) and techniques used for breast reconstruction after mastectomy (9, 10).

Patients were followed from the first to the last consultation at the hospital. If the patient did not return for more than twice the period stipulated in the consultation, the she was considered to have been lost to follow-up. Death was evaluated based on its cause. We considered death from cancer to be the presence of death related to breast cancer. Based on this definition, we examined overall survival (OS) and cancer-specific survival (CSS). The last date of patient evaluations was 29/09/2022.

In the statistical analysis, descriptive statistics were performed for categorical and continuous variables (**Table 1**). Continuous numbers were reported by means and standard deviation (± SD). We also sought to compare potential factors associated with the performance of OBS. The chi-square test was used to compare categorical variables; when there were fewer than five patients in a category, Fisher’s test was performed. For continuous variables, a normality test was performed, and the Mann−Whitney U test was performed for non-normally distributed variables. For the variables associated with OBS, logistic regression was performed to evaluate the impact of each variable on the final result (**Supplementary Table 1**). The Kaplan–Meier method was used to analyse OS and CSS, and the log-rank method was used to evaluate the impact of OBS on survival. Differences were considered significant for p values <0.05. IBM SPSS® for Mac® was used for data collection, tabulation and all statistical analyses.

## Results

During the study period, 85 women with PDB were evaluated. The mean age was 52.2± 13.3 years. Most patients were treated after 2010 (87.0%), and the majority were aged between 40-59 years (56.5%). A minority exclusively had PD (7.1%), and the other cases included DCIS (18.8%), invasive carcinoma (57.6%) and DCIS with invasive carcinoma (16.5%). From a clinical perspective, 58.8% had visible areolar disease, and 57.6% had a palpable tumour. PDB was unilateral in all patients, with a higher frequency on the right side (58.8%), despite the low presence of bilateral breast cancer (4.7%). The mean total size of the tumours was 4.1±3.3 cm. With respect to clinical stage, 27.1% had *in situ* disease, and one patient had metastatic disease at diagnosis (1.2%). With respect to the molecular subtype, HER2-overexpressed tumours represented50.6% of cases, followed by HER2-positive Luminal B tumours (22.9%).

**Figure 1:**
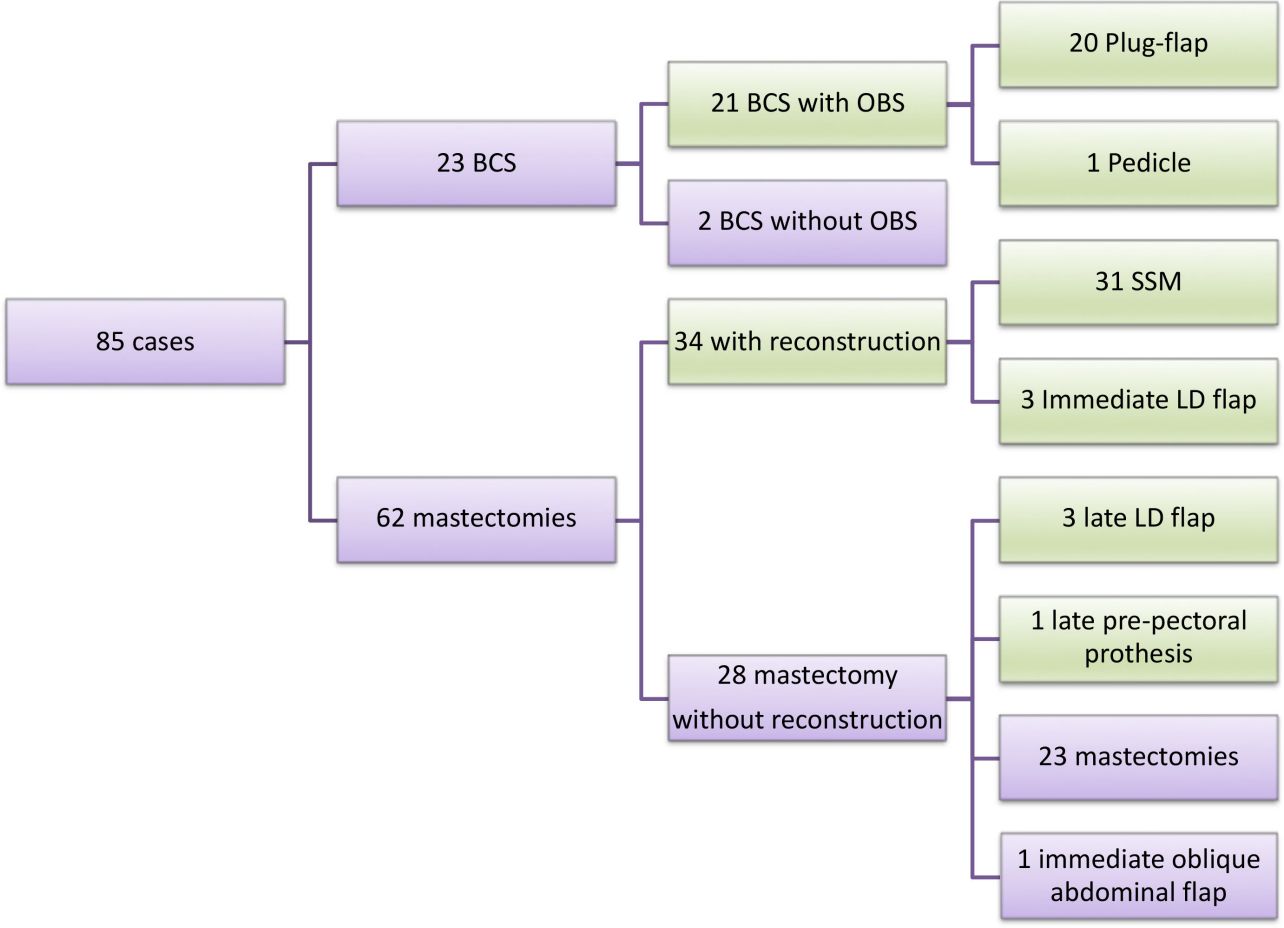
Surgery flowchart. OBS= oncoplastic breast surgery; BCS= breast conserving-surgery; LD= latissimus dorsi. Green = OBS; Purple for all group and non-OBS.

**Figure 2:**
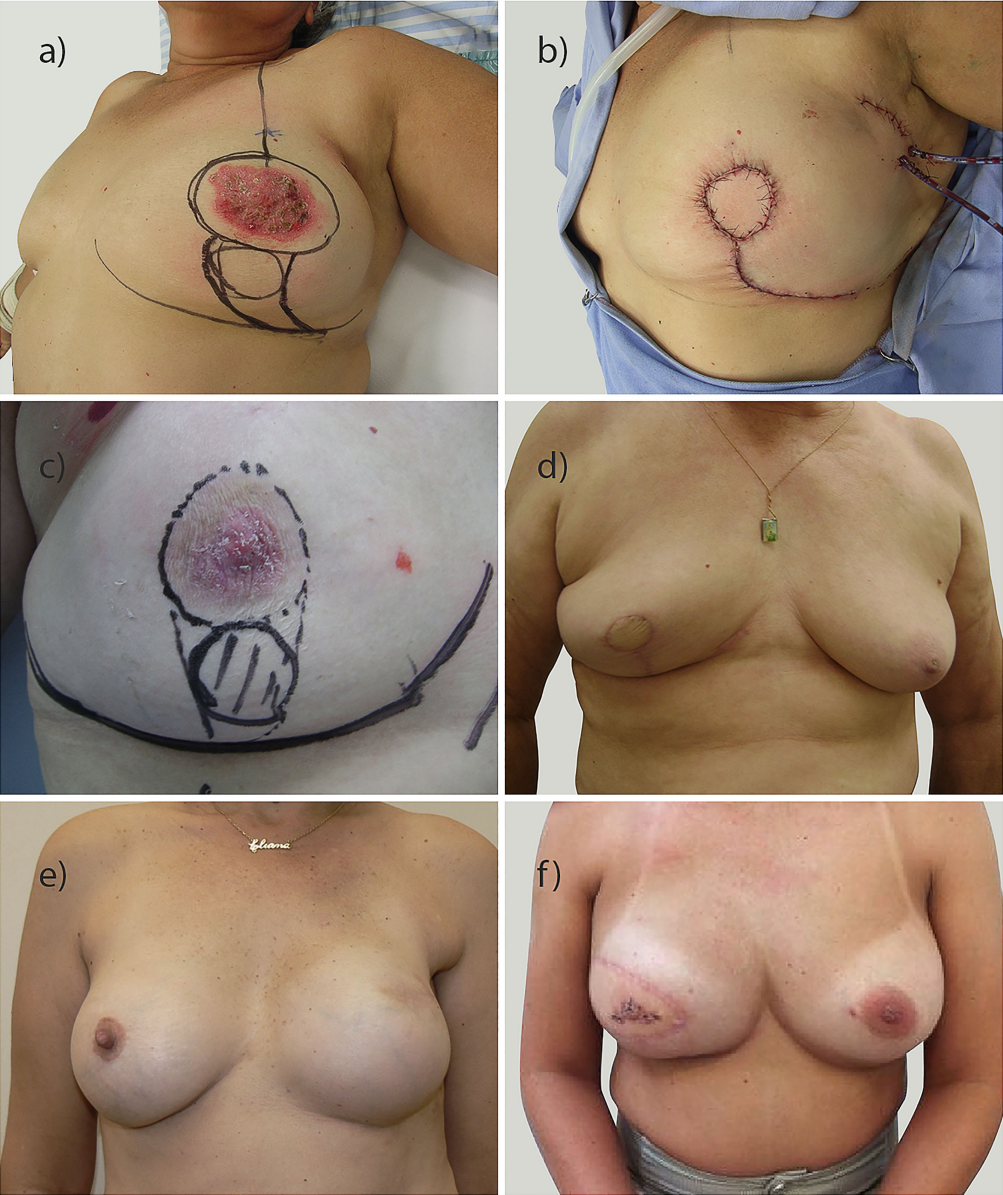
Examples of breast oncoplastic surgery performed. **(A–D)** conservative breast surgery with different plug-flap techniques; **(E)** reconstruction with prosthesis; **(F)** reconstruction with latissimus dorsi.

In the evaluation of surgical treatment, 23 patients (27.0%) underwent BCS. Among those who underwent mastectomy (73.0%; n=62), 54.8% (n=34) underwent immediate reconstruction, typically with submuscular breast prostheses (n=31). Late reconstruction was performed in four patients (6.4%), three with latissimus dorsi and prosthesis (n=3), and one with sufficient skin, where pre-pectoral prosthesis was placed. Of the patients who underwent primary mastectomy without reconstruction, one required the use of the external oblique muscle for skin closure. Because external oblique muscle was used for skin closure, we not considered it as OBS. Overall, 27.0% (n=23) of the patients underwent BCS, and the majority underwent oncoplastic surgery (20 plug-flap, 1 pedicle). All patients had pathologically free margins. Thus, evaluating all surgeries performed (final results), OBS was performed in 69.4% (n=59) of the patients, and of these, 16 (27.2%) were symmetrized to the contralateral breast. The flowchart of the surgical techniques performed is presented in **Figure 1**, and the main types of surgery performed are presented in **Figure 2**.

In the evaluation of factors related to OBS (**Table 1**), an association was found with age and clinical stage at diagnosis. However, in the multivariate analysis, only age was associated with OBS (p = 0.035); the use of OBS was lowest in patients over 70 years of age (**Supplementary Table 1**) and highest in patients in the 40-49–years age group (p=0.002), with an odds ratio of 3.22 [CI 3.39-184.50].

With regard to axillary surgical treatment, 41.2% underwent axillary lymphadenectomy. Adjuvant radiotherapy was performed for 60% (51) of patients, and fossa radiotherapy was performed for 3.5% (3) of patients. Due to the association with invasive disease, 57.6% (49 patients) underwent chemotherapy using various regimens.

For the patients undergoing chemotherapy, 16 were treated with a neoadjuvant regimen. Patients submitted to neoadjuvant chemotherapy had lower rate of OBS (17.1%- present versus 39.1%-absent). Trastuzumab was used in 30 patients (35.3%), primarily as an adjuvant therapy. Hormone therapy was used in 37 patients (43.5%), and tamoxifen (27.1%, n=23) was the primary hormonal medication.

In the mean follow-up period of 71.2± 43.3 months, all patients were followed. At the end of follow-up, 81.2% (n=69) of patients were alive without cancer, 1 (1.2%) developed lung metastasis, 10.6% (n=9) died secondary to disease progression, and 7.1% (n=6) died of non-cancer-related causes. Four patients experienced local recurrence, including patients treated with a variety of different surgical techniques (BCS-OBS, mastectomy without reconstruction, mastectomy with prosthesis, immediate mastectomy with latissimus dorsi reconstruction). The OS and CSS at 120 months were 69.6% and 83.1%, respectively.

OBS did not affect local recurrence or survival (**Tables 1**, **2**). The presence or absence of OBS (**Supplementary Figure 1**) did not affect OS (p=0.558) or CSS (p=0.785). Furthermore, the type of surgery performed did not affect OS or CSS (**Table 2**; **Supplementary Figure 1**).

**Table 1:** Characteristic of the groups related to Oncoplastic Breast Surgery for Paget disease.

Variable	Category	OBS absent	OBS present	Total	p
Age	mean + SD	58.0 ± 15.9	49.0 ± 11.7	52.2 ± 13.3	0.014
Total tumor size	mean + SD	2.2 ± 1.6	5.0 ± 4.0	4.1 ± 3.3	0.548
Follow up	mean + SD	66.5 ± 39.2	71.9 ± 45.3	71.2 ± 43.3	0.932
Age range	< 40	4	8	12 (14.1)	0.01
	40-49	3	25	28 (32.0)	
	50-59	7	13	20 (23.5)	
	60-69	6	11	17 (20.0)	
	> 70	6	2	8 (9.4)	
Treatment period	2000-2009	4	7	11 (12.9)	0.737
	2010-2013	10	18	28 (32.9)	
	2014-2017	4	11	16 (18.8)	
	2018-2021	7	23	30 (35/3)	
Paget	Clinic	15	35	50 (58.8)	0.475
	Pathologic	11	24	35 (41.2)	
Tumor	Palpable	17	32	49 (57.6)	0.475
	Non-palpable	9	27	36 (42.4)	
Laterality	Right	15	35	50 (58.8)	1.000
	Left	11	24	35 (41.2)	
Bilateral tumor	Absent	25	56	81 (95.3)	1.000
	Present	1	3	4 (4.7)	
Histology	PD alone	0	6	2 (2.4)	0.131
	PD+ in situ	6	10	20 (23.5)	
	PD+ invasive	18	31	49 (57.6)	
	PD+ *in situ* + invasive	2	12	14 (16.5)	
Clinical stage	0	5	18	23 (27.1)	0.05
	1	3	17	20 (23.5)	
	2	9	8	17 (20.0)	
	3	8	16	24 (28.2)	
	4	1	0	1 (1.2)	
Molecular	Luminal Her -	2	10	12 (14.5)	0.227
Subtype*	Luminal B Her +	9	10	19 (22.9)	
	Her +	11	31	42 (50.6)	
	Triple negative	4	6	10 (12.0)	
Local recurrence	Absent	25	55	81 (95.3)	1.000
	Present	1	3	4 (4.7)	
Death for cancer	Absent	22	55	76 (89.4)	0.276
	Present	4	5	9 (10.6)	
Death (overall)	Absent	19	51	70 (82.4)	0.215
	Present	7	8	15 (17.6)	

PD, Paget disease; *excluded missing information.

**Table 2:** Survival of patients with PDB in relation to the type of surgery.

Variable	Category	n	60 months	96 months	p (log rank)
Overall OS	–	85	89.1%	72.8%	–
OBS	Absent	30	89.3%	73.7%	0.558
	Present	55	88.3%	72.4%	
Initial OBS	BCS	2	100%	100%	0.675
	BCS + OBS	21	100%	68.6%	
	Mast.	28	88.4%	71.1%	
	Mast.+OBS	34	81.1%	76.1%	
BCS	BCS	2	100%	100%	0.400
	BCS + OBS	21	100%	68.6%	
Mastectomy	Mast.	28	88.4%	71.1%	0.523
	Mast.+OBS	34	81.1%	76.1%	
OBS	BCS	23	100%	74.1%	0.568
	Mast.	28	88.4%	71.1%	
	Mast.+OBS	34	81.1%	76.1%	
Overall CSS	–	85	92.3%	83.1%	–
OBS	Absent	30	100%	81.6%	0.785
	Present	55	91.3%	84.0%	
Initial OBS	BCS	2	100%	100%	0.709
	BCS + OBS	21	100%	90.0%	
	Mast.	28	92.9%	79.4%	
	Mast.+OBS	34	85.9%	80.5%	
BCS	BCS	2	100%	100%	0.400
	BCS + OBS	21	100%	90.0%	
Mastectomy	Mast.	28	92.9%	79.4%	0.523
	Mast.+OBS	34	85.9%	80.5%	
OBS	BCS	23	100%	74.1%	0.568
	Mast.	28	92.9%	79.4%	
	Mast.+OBS	34	85.9%	80.5%	

OS, Overall survival; CSS, cancer specific survival; OBS, oncoplastic breast surgery; Mast., mastectomy.

## Discussion

PDB is a rare entity, generally described in retrospective studies or large databases. Because most series include fewer than one hundred patients (19, 21–23), sample size is limitation; however, we reported 85 cases over 21 years.

Clinically, PDB is characterized by areolar changes such as eczema, desquamation, ulceration or bleeding (24) and a high rate of multifocality (25, 26). PDB has been described separately or in association with carcinoma in situ, invasive breast cancer or both (19, 26, 27), as seen in the current cohort. Because it is a clinical and/or pathological alteration, selected cases that show the clinical characteristics of PDB and subclinical diseases have been presented together in many review articles (28).

Due to the multifocal nature of PDB, simple central resection results in incomplete removal of the lesion in many cases (26). Thus, imaging evaluation is essential in the surgical planning for PDB (29, 30). Mammography typically reveals microcalcifications but can be negative in 50% of cases. The presence of nodulation is generally associated with invasive disease, which can be visualized on mammography and ultrasonography. Magnetic resonance imaging (MRI) of the breast, in turn, assists in the evaluation of new findings, and PDB is currently considered one of the indications for MRI (29, 30). However, its usefulness in radical surgical treatment, i.e., mastectomy, is unknown.

In recent years, with a better understanding of the disease, most patients with PDB and HER 2 expression (26), were submitted to targeted therapies. However, this association has not yet been thoroughly evaluated in the literature.

In previous studies, choices of surgical treatment have been limited to mastectomy or BCS through central quadrantectomy (19, 21, 22), which requires free surgical margins and radiotherapy (19, 30, 31). Depending on the multifocality and extension of the lesion, mastectomy is necessary (32–34). In general, the BCS rate is lower than that of mastectomy, ranging from 10% to 38% (18, 21–23, 27, 33), although one study reported a BCS rate of 60% (19). Past articles reported the feasibility of BCS without reference to the technique (21, 22, 30), although these studies were published when BCS was commonly performed with spindle incisions or purse-string sutures (24).

The surgical treatment of breast cancer has become more complex with the addition of oncoplastic surgery techniques (5), which require adequate treatment planning based on the tumor/breast volume ratio, the presence of ptosis and the tumor location (1, 5, 17, 35). In this regard, due to the preferential central location of PDB, central quadrant resection methodologies have become of great importance in preoperative planning (14, 17, 35, 36). Generally, the Grisotti technique, inferior pedicle reduction or inverted T resection is used (17, 37). The nipple-areola complex (NAC) is resected, and in its place, the tissue can be sutured or the NAC can be replaced by a circumferential island of skin that will be tattooed in the future (14, 37). Specific techniques (**Supplementary Figure 2**), including the Grisotti technique (17), mammoplasty, glandular remodeling (14, 36), and geometric compensation (38), allow the skin total circumference to be created and replace in the local of areola, or when it is difficult, the use of half-moon technique (superoinferior or mediolateral local flaps). Other repair possibilities include the use of locoregional skin flaps (15) and the latissimus dorsi myocutaneous flap for central filling (16, 20). Few articles have focused on the conservative oncoplastic treatment of PDB (15, 16, 24), and few studies have described approaches to reconstruct the central region (16, 17, 19, 36). Despite the limited number of cases in our study, the present work includes one of the largest series of PDB patients undergoing OBS, with the Grisotti plug-flap technique being preferred (n = 20) when using local flaps. In one case, mammoplasty was performed, and a circular area of skin was preserved to allow the tattooing of an areola.

The American Society of Breast Surgeons (8) defines the OBS term exclusively for techniques associated with breast-conserving surgery, but non-American publications (6, 7, 9, 10, 39) also use this term for breast reconstruction after mastectomy, and we opted to use OBS for both conditions. Patients with PDB who undergo mastectomy typically do not undergo reconstruction. There are only a few articles in the literature reporting on patients with PDB who undergo mastectomy also undergo reconstruction, which can be performed with a prosthesis, as in skin-sparing mastectomy or skin-reducing mastectomy (19, 40, 41), a myocutaneous flap, such as the latissimus dorsi (37), or local flaps (15). In one study of 115 patients, 46 mastectomies (40%) were performed, of which 17 (36.9% of the mastectomies) were skin sparing/skin reducing mastectomies (19). Our sample represents the largest series of PDB patients undergoing breast reconstruction, which was performed immediately in 54.1% (33/61) of the mastectomies, preferably with a prosthesis only, or, in some selected cases, the latissimus dorsi and a prosthesis. The decision to perform latissimus dorsi surgery was based on the desire to achieve a good long-term outcome and on selected patients who potentially would not need radiotherapy. Late reconstruction was performed in 4 of the patients who were initially mastectomized, and the preferred treatment was reconstruction with a latissimus dorsi flap and prosthesis. It should be noted that for one patient who underwent mastectomy, delayed reconstruction was possible with a direct prosthesis without the need for an expander due to excess skin associated with nonperformance of radiotherapy, which provided good local conditions.

OBS surgery represents the last paradigm for surgical treatment of breast cancer, and whether it is performed depends on the indication for surgery as well as several additional factors. The presence of a plastic surgeon or a breast surgeon with knowledge of oncoplastic techniques is fundamental. Breast surgeons are currently improving their techniques, and as time goes by, they have become more skilled in performing these techniques, which has led to the expansion of indications for OBS (42). Although the tumor board discusses case management, the surgery board discusses the surgical indications (43, 44). Our group is composed of six surgeons with experience in performing OBS. Since 2010, all cases have been discussed by the tumor board. The surgeon chooses the type of surgery based on the tumor, patient condition, radiological exams and intraoperative frozen sections. Multiple factors are associated with OBS (45), but the surgeon is not a variable associated with OBS because all surgeons are trained in the approach. Few case series have reported on the use of OBS for PDB (19, 20). Our study includes the largest series of patients with PDB undergoing OBS. There was an apparent selection bias for OBS, as it was more likely to be performed in patients in the 40-49 age group, an age group that has previously been described in the literature as being likely to undergo BCS (45).

We opted to evaluate only conditions related to OBS and local recurrence to ensure the focus of this article is surgery. The local recurrence rate for PDB was low (4.7%), which has been observed in other studies (23, 46). The rate of local recurrence was different following different surgical techniques, but despite these results, OBS was not associated with an increased rate of local recurrence. Another study is underway to evaluate the conditions related to distant recurrence and factors related to survival in PDB.

We sought to present the total extent of the disease, which is the sum of the invasive disease and disease in situ, and the factors influencing surgical treatment. Thus, even with large tumors, OBS was performed in a considerable proportion of patients. In the presence of *in situ* disease, surgical treatment does not affect survival; however, whether patients with invasive disease experience worse (33) or similar survival outcomes after adjustment for different variables (47, 48) remains unknown. Similarly, the presence of a palpable lesion is associated with a worse prognosis (28). These factors are likely influenced by the conditions of the invasive disease. It is worth noting that the association of PDB with the Her2 molecular subtype (26) may be related to a worse prognosis for these patients, but paired case−control studies evaluating this association are needed. In this study, we sought to focus more on the conditions associated with surgery and OBS, which did not influence OS or CSS.

The primary limitation of this study is that it is a retrospective evaluation; however, it is difficult to perform prospective studies of rare diseases and evaluate nonadherence to OBS, as they are based on case selection and patient discussion. Because of the retrospective nature of the analysis, it was not possible to evaluate cosmesis and quality of life in our patients. OBS was shown in the present study to be feasible, and its performance was not associated with local recurrence, nor did it influence survival, thus justifying OBS for PDB.

As surgeons become more experienced in performing OBS, more patients with PDB will undergo OBS. We anticipate future publications on the topic, but we are the first to report a high rate of OBS for PDB.

## Conclusion

OBS improves options related to surgical treatment for patients with PDB without affecting local recurrence or survival rates. To this end, it is necessary to select appropriate cases by means of clinical evaluation and imaging, and surgeons must be aware of the various OBS techniques.

## Data availability statement

The raw data supporting the conclusions of this article will be made available by the authors, without undue reservation.

## Ethics statement

The studies involving human participants were reviewed and approved by Research Ethics Committee from Barretos Cancer Hospital under numbers 657293 and CAAE 31046314.5.0000.5437. Written informed consent for participation was not required for this study in accordance with the national legislation and the institutional requirements.

## Author contributions

RP and RV conceived and wrote the manuscript. RP and IdO evaluated the data. RV supervised the study and performed data analysis. All authors contributed to the article and approved the submitted version.

## Supplementary material

Supplementary Figure 1 Actuarial survival associated with OBS. **(A, B)** Cancer-Specific Survival; **(C, D)** Overall Survival. OBS= oncoplastic breast surgery; Mast.=mastectomy; BCS= breast conserving-surgery.

Supplementary Figure 2 More solutions for breast central disease reconstruction in women with small/medium breasts. **(A)** Single half-moon skin flaps; **(B)** Half-moon flaps associated with reduction mammoplasty technique; **(C)** geometric compensation with areolar resection (Previously published with author authorization).

## References

[1] Silverstein MJ . Radical mastectomy to radical conservation (Extreme oncoplasty): a revolutionary change. J Am Coll Surg (2016) 222(1):1–9. doi: 10.1016/j.jamcollsurg.2015.10.007 26778582

[2] Oliveira-Junior I Haikel RL Vieira RAC . Breast-conserving treatment in oncoplastic times: indications, cosmesis, and quality of life. Mastol (2021) 31(1):e20200040. doi: 10.29289/2594539420200040

[3] Vieira RA Carrara GF Scapulatempo Neto C Morini MA Brentani MM Folgueira MA . The role of oncoplastic breast conserving treatment for locally advanced breast tumors. a matching case-control study. Ann Med Surg (Lond) (2016) 10:61–8. doi: 10.1016/j.amsu.2016.08.001 PMC4983144 27547399

[4] Agrawal A Sibbering DM Courtney CA . Skin sparing mastectomy and immediate breast reconstruction: a review. Eur J Surg Oncol (2013) 39(4):320–8. doi: 10.1016/j.ejso.2012.12.015 23333068

[5] Audretsch W Andree C . Is mastectomy still justified–and if, in which patients? Onkologie (2006) 29(6):243–5. doi: 10.1159/000093477 16770084

[6] Zucca-Matthes G Manconi A da Costa Viera RA Michelli RA Matthes Ado C . The evolution of mastectomies in the oncoplastic breast surgery era. Gland Surg (2013) 2(2):102–6. doi: 10.3978/j.issn.2227-684X.2013.05.03 PMC4115729 25083466

[7] Atiyeh B Dibo S Zgheib E Abbas J . Skin sparing/skin reducing mastectomy (SSM/SRM) and the concept of oncoplastic breast surgery. Int J Surg (2014) 12(10):1115–22. doi: 10.1016/j.ijsu.2014.08.401 25178261

[8] Chatterjee A Gass J Patel K Holmes D Kopkash K Peiris L . A consensus definition and classification system of oncoplastic surgery developed by the American society of breast surgeons. Ann Surg Oncol (2019) 26(11):3436–44. doi: 10.1245/s10434-019-07345-4 30977016

[9] Weber WP Haug M Kurzeder C Bjelic-Radisic V Koller R Reitsamer R . Oncoplastic breast consortium consensus conference on nipple-sparing mastectomy. Breast Cancer Res Treat (2018) 172(3):523–37. doi: 10.1007/s10549-018-4937-1 PMC6245050 30182349

[10] Weber WP Shaw J Pusic A Wyld L Morrow M King T . Oncoplastic breast consortium recommendations for mastectomy and whole breast reconstruction in the setting of post-mastectomy radiation therapy. Breast (2022) 63:123–39. doi: 10.1016/j.breast.2022.03.008 PMC8976143 35366506

[11] Char S Bloom JA Erlichman Z Jonczyk MM Chatterjee A . A comprehensive literature review of patient-reported outcome measures (PROMs) among common breast reconstruction options: what types of breast reconstruction score well? Breast J (2021) 27(4):322–9. doi: 10.1111/tbj.14186 33565192

[12] Losken A Hart AM Chatterjee A . Updated evidence on the oncoplastic approach to breast conservation therapy. Plast Reconstr Surg (2017) 140(5S Advances in Breast Reconstruction):14S–22S. doi: 10.1097/PRS.0000000000003951 29064918

[13] Galimberti V Vicini E Corso G Morigi C Fontana S Sacchini V . Nipple-sparing and skin-sparing mastectomy: review of aims, oncological safety and contraindications. Breast (2017) 34(Suppl 1):S82–4. doi: 10.1016/j.breast.2017.06.034 PMC5837802 28673535

[14] Vieira RAC Zucca-Matthes G Haikel RL . Central quadrantectomy. In: Zucca-Matthes G , editor. Oncoplastic breast surgery: practical application, 1st ed. Portland, Oregon: ESKA publishing (2016). p. 223.

[15] Kijima Y Yoshinaka H Hirata M Nakajo A Arima H Okumura H . Oncoplastic breast surgery combining partial mastectomy with immediate breast reshaping using a keyhole-shaped skin glandular flap for paget’s disease. Surg Today (2014) 44(9):1783–8. doi: 10.1007/s00595-013-0687-1 23925716

[16] Moustafa A Fakhr I . Outcome of different oncoplastic surgical (OPs) techniques for centrally located breast cancer (CLBC). J Egypt Natl Canc Inst (2014) 26(4):203–9. doi: 10.1016/j.jnci.2014.10.003 25467388

[17] Canturk NZ Simsek T Ozkan Gurdal S . Oncoplastic breast-conserving surgery according to tumor location. Eur J Breast Health (2021) 17(3):220–33. doi: 10.4274/ejbh.galenos.2021.2021-1-2 PMC8246052 34263149

[18] Lin CW Chiang MH Tam KW . Treatment of mammary paget disease: a systematic review and meta-analysis of real-world data. Int J Surg (2022) 107:106964. doi: 10.1016/j.ijsu.2022.106964 36309195

[19] Scardina L Di Leone A Magno S Franco A Biondi E Sanchez AM . Paget’s disease of the breast: our 20 years’ experience. Front Oncol (2022) 12:995442. doi: 10.3389/fonc.2022.995442 36483034 PMC9722944

[20] Kijima Y Yoshinaka H Shinden Y Hirata M Nakajo A Arima H . Oncoplastic breast surgery for centrally located breast cancer: a case series. Gland Surg (2014) 3(1):62–73. doi: 10.3978/j.issn.2227-684X.2013.11.01 25083497 PMC4115775

[21] Singh A Sutton RJ Baker CB Sacks NP . Is mastectomy overtreatment for paget’s disease of the nipple? Breast (1999) 8(4):191–4. doi: 10.1054/brst.1999.0025 14731439

[22] Kawase K Dimaio DJ Tucker SL Buchholz TA Ross MI Feig BW . Paget’s disease of the breast: there is a role for breast-conserving therapy. Ann Surg Oncol (2005) 12(5):391–7. doi: 10.1245/ASO.2005.05.026 15915373

[23] Caliskan M Gatti G Sosnovskikh I Rotmensz N Botteri E Musmeci S . Paget’s disease of the breast: the experience of the European institute of oncology and review of the literature. Breast Cancer Res Treat (2008) 112(3):513–21. doi: 10.1007/s10549-007-9880-5 18240020

[24] Markarian S Holmes DR . Mammary paget’s disease: an update. Cancers (Basel) (2022) 14(10):2422–31. doi: 10.3390/cancers14102422 35626023 PMC9139704

[25] Sandoval-Leon AC Drews-Elger K Gomez-Fernandez CR Yepes MM Lippman ME . Paget’s disease of the nipple. Breast Cancer Res Treat (2013) 141(1):1–12. doi: 10.1007/s10549-013-2661-4 23929251

[26] Kothari AS Beechey-Newman N Hamed H Fentiman IS D’Arrigo C Hanby AM . Paget disease of the nipple: a multifocal manifestation of higher-risk disease. Cancer (2002) 95(1):1–7. doi: 10.1002/cncr.10638 12115309

[27] Zheng S Song QK Zhao L Huang R Sun L Li J . Characteristics of mammary paget’s disease in China: a national-wide multicenter retrospective study during 1999-2008. Asian Pac J Cancer Prev (2012) 13(5):1887–93. doi: 10.7314/APJCP.2012.13.5.1887 22901142

[28] Piekarski J Jeziorski A Baklinska M Szymczak W Zadrozny M Berner J . Patients with paget disease of nipple and with palpable mass in breast have unfavorable prognosis. J Exp Clin Cancer Res (2004) 23(1):33–7. 15149148

[29] Lim HS Jeong SJ Lee JS Park MH Kim JW Shin SS . Paget disease of the breast: mammographic, US, and MR imaging findings with pathologic correlation. Radiographics (2011) 31(7):1973–87. doi: 10.1148/rg.317115070 22084182

[30] Helme S Harvey K Agrawal A . Breast-conserving surgery in patients with paget’s disease. Br J Surg (2015) 102(10):1167–74. doi: 10.1002/bjs.9863 26175231

[31] Polgar C Orosz Z Kovacs T Fodor J . Breast-conserving therapy for paget disease of the nipple: a prospective European organization for research and treatment of cancer study of 61 patients. Cancer (2002) 94(6):1904–5. doi: 10.1002/cncr.10405 11920556

[32] Fu W Mittel VK Young SC . Paget disease of the breast: analysis of 41 patients. Am J Clin Oncol (2001) 24(4):397–400. doi: 10.1097/00000421-200108000-00019 11474272

[33] Chen CY Sun LM Anderson BO . Paget disease of the breast: changing patterns of incidence, clinical presentation, and treatment in the U. S Cancer (2006) 107(7):1448–58. doi: 10.1002/cncr.22137 16933329

[34] Yasir M Khan M Lotfollahzadeh S . Mammary paget disease. Treasure Island (FL: StatPearls (2022). 33085375

[35] Gainer SM Lucci A . Oncoplastics: techniques for reconstruction of partial breast defects based on tumor location. J Surg Oncol (2011) 103(4):341–7. doi: 10.1002/jso.21672 21337569

[36] Pasta V D’Orazi V Merola R Frusone F Amabile MI De Luca A . Oncoplastic central quadrantectomies. Gland Surg (2016) 5(4):422–6. doi: 10.21037/gs.2016.04.01 PMC4971343 27563564

[37] Farouk O Attia E Roshdy S Khater A Senbe A Fathi A . The outcome of oncoplastic techniques in defect reconstruction after resection of central breast tumors. World J Surg Oncol (2015) 13:285. doi: 10.1186/s12957-015-0688-1 26409877 PMC4584018

[38] Franca FC de Oliveira-Junior I Morgan AM Haikel RL da Costa Vieira RA . Breast-conserving surgery with the geometric compensation/split reduction technique. indications, oncologic safety and cosmesis. a cohort series and systematic review of the literature. Surg Oncol (2022) 44:101839. doi: 10.1016/j.suronc.2022.101839 35994978

[39] Kolacinska A . How can we improve education of breast surgeons across Europe? Chirurgia (Bucur) (2017) 112(4):365–6. doi: 10.21614/chirurgia.112.4.365 28862111

[40] Fushimi A Kinoshita S Kudo R Takeyama H . Incidental discovery of follicular lymphoma by sentinel lymph node biopsy and skin-sparing mastectomy for paget’s disease associated with invasive breast cancer. J Surg Case Rep (2019) 2019(1):rjz008. doi: 10.1093/jscr/rjz008 30697416 PMC6344924

[41] Pu Q Zhao Q Gao D . Local recurrence of mammary paget’s disease after nipple-sparing mastectomy and implant breast reconstruction: a case report and literature review. World J Surg Oncol (2022) 20(1):285. doi: 10.1186/s12957-022-02746-4 36064544 PMC9444701

[42] Vieira RAC Matthes AGZ Michelli RAD Ribeiro GFP Mendonça MLH Bailão-Junior A . Oncoplastic surgery and breast surgeon training. Rev Bras Mastol (2010) 20(2):66–70.

[43] Franceschini G Mason EJ Di Leone A Scardina L Masetti R . Multidisciplinary management of mammary paget’s disease: recommendations to optimize oncological and aesthetic outcomes. Ann Ital Chir (2022) 93:481–2. 36196555

[44] Hudson-Phillips S Cox K Patel P Al Sarakbi W . Paget’s disease of the breast: diagnosis and management. Br J Hosp Med (Lond) (2023) 84(1):1–8. doi: 10.12968/hmed.2022.0439 36708338

[45] de Oliveira-Junior I da Silva IA da Silva FCB da Silva JJ Sarri AJ Paiva CE . Oncoplastic surgery in breast-conserving treatment: patient profile and impact on quality of life. Breast Care (Basel) (2021) 16(3):243–53. doi: 10.1159/000507240 PMC8248771 34248465

[46] Li YJ Huang XE Zhou XD . Local breast cancer recurrence after mastectomy and breast-conserving surgery for paget’s disease: a meta-analysis. Breast Care (Basel) (2014) 9(6):431–4. doi: 10.1159/000368431 PMC4317677 25759627

[47] Yao Y Sun L Meng Y Zhuang Y Zhao L Yu Q . Breast-conserving surgery in patients with mammary paget’s disease. J Surg Res (2019) 241:178–87. doi: 10.1016/j.jss.2019.03.025 31026796

[48] Wu Q Ding X Li J Sun S Zhu S Wu J . Surgical treatment in paget’s disease with invasive ductal carcinoma: an observational study based on SEER. Sci Rep (2017) 7:45510. doi: 10.1038/srep45510 28422090 PMC5395813

