# Supplementary figures and images for: Oncoplastic surgery for Paget’s disease of the breast
Source: Front Oncol. 2023 May 17;13:1151932. doi: 10.3389/fonc.2023.1151932 (PMC10231681; doi:10.3389/fonc.2023.1151932)

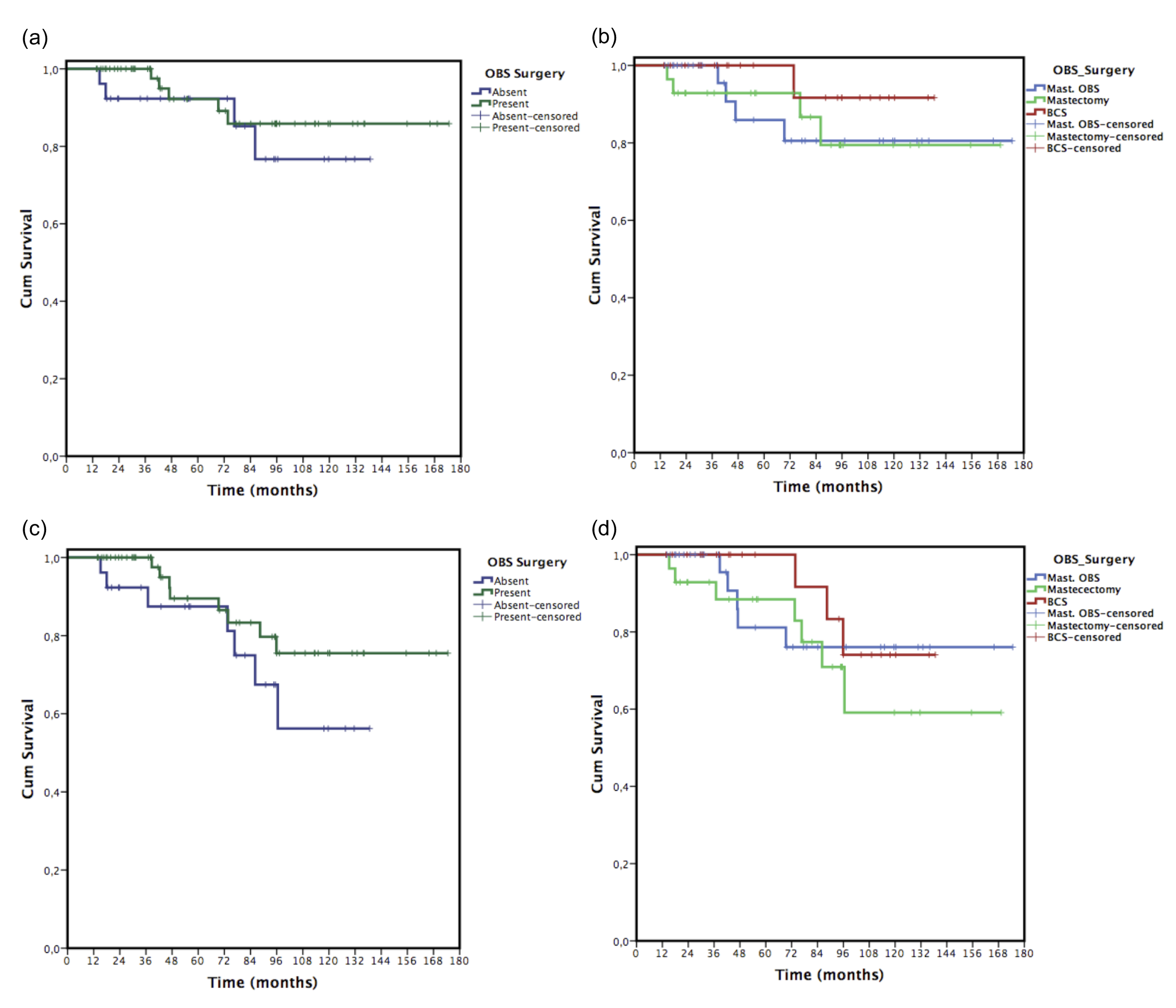

Supplement: Supplementary Figure 1 — Actuarial survival associated with OBS. (A, B) Cancer-Specific Survival; (C, D) Overall Survival. OBS= oncoplastic breast surgery; Mast.=mastectomy; BCS= breast conserving-surgery. [file Image_1.tif]

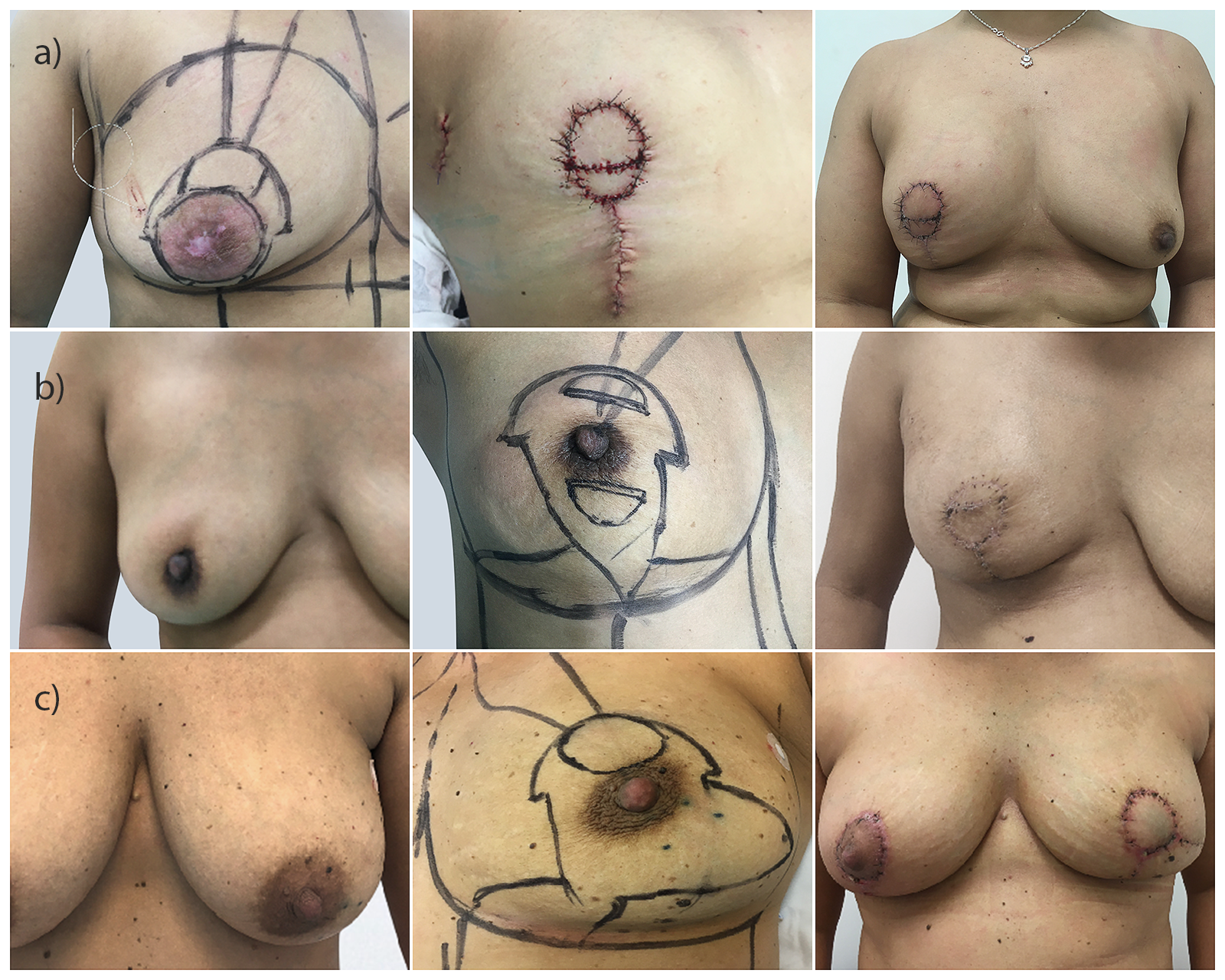

Supplement: Supplementary Figure 2 — More solutions for breast central disease reconstruction in women with small/medium breasts. (A) Single half-moon skin flaps; (B) Half-moon flaps associated with reduction mammoplasty technique; (C) geometric compensation with areolar resection (Previously published with author authorization). [file Image_2.tif]
