# Supplementary material for: Oncoplastic surgery for Paget’s disease of the breast
Source: Front Oncol. 2023 May 17;13:1151932. doi: 10.3389/fonc.2023.1151932 (PMC10231681; doi:10.3389/fonc.2023.1151932)
Supplement: Supplementary file 3 [file Table_1.docx]

**Supplementary Table 1.**

Multivariate analysis related to Oncoplastic Surgery for Paget disease

|  | Analysis | OR | CI | p | p |
| --- | --- | --- | --- | --- | --- |
| Age range | > 70 | 1.00 | reference |  | 0.035 |
|  | < 40 | 1.79 | 0.01-44.35 | 0.079 |  |
|  | 40-49 | 3.22 | 3.39-184.50 | 0.002 |  |
|  | 50-59 | 1.72 | 0.88-35.27 | 0.068 |  |
|  | 60-69 | 1.70 | 0.84-36.20 | 0.076 |  |

OR= odds ration; CI= confidence interval
